# A historical argument for regulatory failure in the case of Primodos and other hormone pregnancy tests

**DOI:** 10.1016/j.rbms.2018.09.003

**Published:** 2018-10-23

**Authors:** Jesse Olszynko-Gryn, Eira Bjørvik, Merle Weßel, Solveig Jülich, Cyrille Jean

**Affiliations:** a Department of History and Philosophy of Science, University of Cambridge, Cambridge, UK; b Department of Community Medicine and Global Health, Faculty of Medicine, University of Oslo, Oslo, Norway; c Department of History, Ernst-Moritz-Arndt University Greifswald, Greifswald, Germany; d Department of History of Science and Ideas, Uppsala University, Uppsala, Sweden; e Sciences Po History Centre, Paris, France

**Keywords:** activism, birth defects, drug safety, health policy, pregnancy, teratogenicity

## Abstract

The drug Primodos and other hormone pregnancy tests (HPTs) remained on the British market for about a decade after they were first implicated, in 1967, as a possible cause of birth defects. In November 2017, an expert working group (EWG) set up by the Medicines and Healthcare Products Regulatory Agency (MHRA) concluded against such an association. However, it was explicitly ‘not within the remit of the EWG to make formal conclusions or recommendations on the historical system or regulatory failures’, a situation that has left many stakeholders dissatisfied. Placing the question of a teratogenicity to one side, this article takes a more contextual and comparative approach than was possible under the auspices of MHRA. It asks why an unnecessary and possibly even harmful drug was allowed to remain on the British market when a reliable and perfectly safe alternative existed: urine tests for pregnancy. Based on archival research in several countries, this article builds a historical argument for regulatory failure in the case of HPTs. It concludes that the independent review which campaigners are calling for would have the potential to not only bring them a form of closure, but would also shed light on pressing issues of more general significance regarding risk, regulation and communication between policy makers, medical experts and patients.

In 1967, Dr. Isabel Gal, a Surrey-based paediatrician, suggested in a brief letter to *Nature* that hormonal pregnancy tests (HPTs) might be causing spina bifida in the children of mothers who had taken the drugs while pregnant (Gal et al., 1967). Primodos, the first HPT, was launched in 1950 by the West German pharmaceutical company Schering AG (today Bayer). It was marketed to doctors both as a treatment for menstrual irregularities and as a convenient test for early pregnancy. Like the other HPTs that followed, Primodos functioned diagnostically by inducing menstrual-like bleeding in non-pregnant women (a ‘negative’ result); no bleeding implied pregnancy. In Britain, Schering took Primodos off the market in 1978 amidst allegations that HPTs caused miscarriage and a range of birth defects.^1^ At around the same time, a group of parents formed the Association for Children Damaged by Hormone Pregnancy Tests (ACDHPT) to take civil action against Schering. The action was discontinued in 1982 but on terms that left the plaintiffs free to proceed again pending further evidence that Primodos caused birth defects.

A few years ago, the discovery of previously inaccessible archival records in London and Berlin revitalized the long-dormant campaign. Led by patient-activist Marie Lyon, ACDHPT is today supported by Gregory Abrams Davidson Solicitors and an All-Party Parliamentary Group chaired by Labour MP Yasmin Qureshi. To date, the archival evidence has been used most effectively by investigative journalist Jason Farrell in ‘Primodos: the Secret Drug Scandal’, a Sky News documentary that, when screened in Parliament on 21 March 2017, reignited calls for a ‘public inquiry into the alleged failure of the regulator at that time to protect public safety’ (Alton, 2017).

Previously, the Commission on Human Medicines (CHM), a committee of the Medicines and Healthcare Products Regulatory Agency (MHRA), had established an expert working group (EWG) to forensically review the medical case for the teratogenicity, or not, of HPTs. The EWG's final report, published in November 2017, concluded that the scientific evidence did ‘not support a causal association between the use of HPTs, such as Primodos, during early pregnancy and adverse outcomes’ (Commission on Human Medicines, 2017: 100). However, it was explicitly ‘not within the remit of the EWG to make formal conclusions or recommendations on the historical system or regulatory failures’ (Commission on Human Medicines, 2017: 2), a situation that has left many stakeholders dissatisfied (Gulland, 2017).

A main contention is that by 1967, when Gal published against Primodos in *Nature*, a convenient, widely available and non-invasive alternative existed: urine tests for pregnancy. Several countries were quick to take regulatory action prohibiting the use of HPTs, and there is a sense that Britain could have taken more decisive action sooner. Even if the evidence against Primodos was weak or inconclusive, as many experts believed, the availability of urine tests meant that removing HPTs from the market would not have caused any harm.^2^ Primodos was no life-extending cancer drug, for which it might be sensible from a regulatory perspective to accept low or even high risks if they were sufficiently offset by the benefits. Rather, as experts also agreed early on, it was a redundant, unnecessary and possibly even harmful drug for which a definitely harmless alternative existed. So why was it allowed to remain on the British market for so many years?

Our aim in this article, which began at a conference in Cambridge in January 2017, is to investigate the nationally specific and even idiosyncratic factors that contributed to the British Government's decision to leave Primodos and other HPTs on the market. Placing the still open question of teratogenicity to one side, we take a more contextual and comparative approach than was possible under the auspices of MHRA.^3^ Based on archival research in several countries, we build a historical argument for apparent regulatory failure on the part of CHM's predecessor organizations: the Committee on Safety of Drugs (1963–1970) and the Committee on the Safety of Medicines (1970–2005). We do so chiefly by reconstructing the British timeline, and comparing this with parallel developments in Norway and other countries that followed a more precautionary line. We begin by reviewing the history of pregnancy testing to clarify the chronology of availability of more or less risky alternatives.

## Marketing pills as pregnancy tests

Today's home tests are ubiquitous retail objects that can be purchased cheaply from any pharmacy, supermarket or online. They are highly reliable from the day of a missed period, and work by detecting the presence of human chorionic gonadotrophin (hCG) in a woman's urine. However, pregnancy testing was not always so easy. The Primodos decades (1950s–1970s) saw three major and, to some extent, overlapping regimes: bioassays (1929–1964), immunoassays (since 1962) and home tests (since 1971). These corresponded to the mass adoption of new diagnostic technologies, services and supply chains, as well as to changing social arrangements between women, doctors and pharmacists. To simplify a complicated story, pregnancy testing began as a laboratory service under medical control that was typically reserved for differential diagnosis in ‘pathological’ cases. From the mid 1960s, women increasingly gained access to ‘social’ pregnancy testing, not as patients but as consumers (Olszynko-Gryn, 2014b).

To go into slightly more detail, the Aschheim-Zondek ‘mouse’ test for early pregnancy, a German innovation, was adopted in Britain in 1929 as the first reliable bioassay for hCG (Olszynko-Gryn, 2014a). Each test involved injecting several mice with a woman's urine, then killing and dissecting the mice to observe the presence, or not, of characteristic ovarian changes induced by the hormone. Mice (and rabbits) were supplanted by reusable toads as diagnostic services ramped up in the late 1940s under the new National Health Service (NHS) (Gurdon and Hopwood, 2000). The Dutch pharmaceutical company Organon launched Pregnosticon, the first immunoassay, in 1962 and most laboratories abandoned the use of animals shortly thereafter. Predictor, the first reliable home test, debuted in 1971. It resembled a small chemistry set and was not an overnight commercial success. Only after Unilever launched Clearblue One Step in 1988 did a younger generation of women embrace self-testing as the new normal (Olszynko-Gryn, 2017b).

Today, it may be difficult to believe that doctors ever prescribed pills as pregnancy tests. However, attempts to develop a ‘therapeutic’ or ‘clinical’ test for pregnancy that acted on the patient herself and so did not depend on a laboratory go back at least to the 1910s (Henriksen, 1941). Invasive pregnancy tests included eye-drop tests as well as skin reaction tests inspired by those for diphtheria, scarlet fever and tuberculosis, as well as hay fever and other allergies (Jackson, 2007, Smith, 2015). Other methods, including the prostigmine test for early pregnancy, were said to function as Primodos later would, namely, by inducing menstrual bleeding in non-pregnant women alone (Soskin et al., 1940). As with parallel attempts to develop a simple colour-change reaction in a test tube, most invasive tests prior to 1950 were deemed insufficiently accurate for clinical application and were not widely adopted.

HPTs emerged as the result of research not on pregnancy diagnosis, but on the treatment of amenorrhoea, a common condition that was often associated with infertility, but could also be caused by pregnancy. Bernhard Zondek, the famous co-inventor of the celebrated mouse test, pioneered the combined injection of synthetic oestrogen and progesterone in the treatment of amenorrhoea in Palestine in the early 1940s (Novick, 2014). His article in the *Journal of the American Medical Association* became a touchstone for what became known as ‘Zondek's method’ of non-surgical curettage (Zondek, 1942). After World War II, pharmaceutical companies began marketing hormone treatments for amenorrhoea, not directly to women but to their (then almost always male) gynaecologists (Oudshoorn, 1994). New forms of synthetic sex hormones enabled the crucial shift from injections to better tolerated tablets in the late 1950s. These significantly opened up the market and cleared the way for oral contraceptives.

Primodos, as indicated by the German trade name ‘*Duo*gynon’, innovatively combined Schering's two leading gynaecological products: Proluton and Progynon. As such, it contained the same mixture of synthetic sex hormones that would later constitute Anovlar, Schering's commercially successful oral contraceptive (Thoms, 2014).^4^ Schering, an undisputed leader in hormone research and marketing (Gaudillière, 2005), initially presented the new drug primarily as a treatment for amenorrhoea. Advertisements in Schering's in-house journal show that Duogynon's secondary function as a test for early pregnancy began almost as an afterthought in 1950, and then increased in prominence to become the dominant indication a few years later. By 1960, Schering promoted Duogynon/Primodos, now in tablet form, exclusively for pregnancy testing. Other companies soon followed suit with competing products, notably Roussel's Amenorone Forte, but Schering continued to lead the market in most countries. In contrast to the toad test, which was not considered effective until 2 weeks after a missed period, HPTs were indicated for use on the first day of amenorrhoea.

In postwar Britain, the rise of Primodos coincided with a significant increase in demand for pregnancy testing. One country doctor, for example, ordered pregnancy tests for only 1.3% of his female patients in the late 1940s, a proportion that had increased to 38.8% by the late 1970s (Oakley, 1984: 230). Meanwhile, doctors' requests for all types of laboratory investigations, including pregnancy tests, doubled between 1961 and 1971, straining a public health system that was facing a major financial crisis by the mid 1970s (Olszynko-Gryn, 2017a). In the 1950s, however, supply from laboratories seems to have plateaued; expansion was constrained by overhead costs and infrastructure requirements for animals, housing, technicians and general maintenance (Olszynko-Gryn, 2014b: 146). As laboratories failed to keep up with increasing demand, Schering and other companies captured part of a lucrative and expanding market.

Primodos and other HPTs were initially marketed as plausibly advantageous over the toad test and, before the thalidomide tragedy (1957–1961), neither consumers nor experts were accustomed to associating prescription drugs with risk of harm to the fetus (Clow, 2003). Thalidomide may have raised concerns about the permeability of the placental barrier (Martin and Holloway, 2014), but because the pregnant body ordinarily produces high levels of sex hormones, the comparably small dosages in HPTs were widely regarded as harmless. Progesterone therapy was widely used in the 1950s to prevent miscarriage, and many doctors believed that HPTs, far from causing harm, would even ‘help implant the ovum properly’ (Anon., 1960). Marketing literature reported that anxious patients found the physiological certainty of uterine bleeding more reassuring than a laboratory report, which could be mistaken (Squibb, 1962: 76–77). Primodos was, moreover, a test that the woman took home to perform privately, the result known to her alone. HPTs thus conferred some of the discretion that Predictor and other self-testing kits later would.^5^

Some experts, however, expressed misgivings early on. In 1956, London physiologist Hubert Britton, having received ‘with dismay’ a ‘brochure from a drug firm’ advertising the use of synthetic sex hormones as pregnancy tests, voiced concern in the *British Medical Journal* (*BMJ*) that such tests could potentially ‘upset the delicate hormonal balance’ of pregnancy and provoke miscarriage or even damage the embryo at its ‘most susceptible’ stage of development (Britton, 1956). Two years later, Birmingham geneticist John Edwards wrote in the *British Journal of Preventive and Social Medicine* that the ‘widely advertised’ HPTs could provide ‘the type of insult which is likely to cause foetal malformations, and would often be administered at a stage in pregnancy when it might initiate malformations of the central nervous system’ (Edwards, 1958: 128). In 1961, soon after Schering's British marketing blitz, *Woman* magazine warned against the unknown risks of HPTs and promoted the toad, not the tablets, as a ‘modern scientific achievement’ (Seaward, 1961).

Hospital doctors had access to in-house laboratories and so did not generally prescribe HPTs, but many general practitioners (GPs) embraced Primodos, especially if they were overworked and had limited access to alternatives. For instance, a pair of Bristol doctors, who provided antenatal care to 7500 patients in 1960, regarded the collection and transport of urine as a ‘considerable inconvenience to an already busy person’. Instead of the ‘cumbersome’ and ‘lengthy’ toad test, they decided to give Primodos to ‘all women’ who had amenorrhoea of short duration, excluding those who were ‘clearly pregnant’ (Higgens and Sadler, 1960).

Organon's Pregnosticon put paid to the use of animals in pregnancy diagnosis, and was perceived by Schering researchers as a commercial threat to Duogynon (Ufer, 1962: 9). From 1965, small commercial laboratories in London used Pregnosticon or one of the other immunoassays already on the market to serve women not as ‘patients’ but as ‘clients’. Most charged £2 for a pregnancy test, or the equivalent of a week's rent for a student in Leeds in the late 1960s (Olszynko-Gryn, 2017a). Primodos cost only 5 shillings, one-eighth the cost of a urine test. As prescription drugs, HPTs were initially reimbursable on the NHS, and many patients were sent home with free samples. The Ministry of Health placed Pregnosticon on central supply to hospital pathology departments in 1967 (Olszynko-Gryn, 2014a: 229), but access to laboratory services was distributed unevenly outside major cities so prescribing tablets was often cheaper and quicker than ordering a urine test. Hence, geographic variation in income levels and the uneven availability of alternatives helps to explain why a *Sunday Times* survey found that 10 of 12 doctors in South Wales still used HPTs in 1975 (Gillie, 1975).

## The early-warning system

Pharmaceutical companies faced few obstacles when bringing drugs to market in the 1950s. The thalidomide tragedy not only solidified the conception of the placental barrier as dangerously permeable, but also transformed teratology – the science of birth defects – into a much sought-after specialism and motivated the extension of national systems of pharmacovigilance to include the risk of potentially teratogenic drugs (Al-Gailani, 2014, Dron, 2016, Löwy, 2017, Reagan, 2010). In Britain, the tragedy brought into public view a debate that was already quietly underway in the corridors of power, but legislative reform did not come into effect for another decade, with the implementation of the 1968 Medicines Act in 1971 (Abraham, 2009).

The Medicines Division, a national drug regulatory agency within the Department of Health and Social Services (DHSS), was formed in 1971 to administer the Medicines Act. It comprised of a full-time scientific staff to review industry-furnished data on new drugs, and empowered to permit (or deny) approval to those drugs for the British market. The Act applied to new drugs alone; thousands of ‘old’ drugs, including Primodos, could continue to be used in the NHS without further review. In 1975, in response to a European Economic Community directive, the DHSS established a committee to assess the safety and efficacy of old drugs, a task completed in 1990. In addition to public safety and cost saving for the NHS, industry interests were, from the start, an important concern of government officials, who consulted closely with pharmaceutical companies (Abraham, 2009).

In 1963, amidst the fallout from thalidomide, the Government established the Committee on Safety of Drugs (CSD), known colloquially as the ‘Dunlop Committee’ after its first chair Sir Derrick Dunlop, a prominent Scottish physician and pharmacologist. The CSD was tasked with reviewing data submitted by pharmaceutical companies, and with advising manufacturers and the Government on whether new drugs had been adequately tested for market. It had no legal powers and depended on voluntary cooperation from industry. By the end of 1965, it consisted of a small team of six doctors, three pharmacists and a modest administrative staff. Its members were not employed by pharmaceutical companies, but were permitted to have financial interests, such as shareholdings or research grants. The review process was confidential and thus protected from public scrutiny. It was also designed to be rapid so as not to needlessly delay or prevent the introduction of potentially beneficial drugs (Abraham, 1995, Tansey and Reynolds, 1997).

The CSD was divided into three subcommittees on toxicity, clinical trials and adverse reactions, the latter of which was first chaired by Oxford professor Leslie Witts. Dr. William ‘Bill’ Inman joined the CSD as Senior Medical Officer and Medical Assessor for the Witts Subcommittee in 1964. Previously, he had battled polio as a medical student at Cambridge before acting as Medical Adviser to Imperial Chemical Industries, the company his father had cofounded in 1926. He was later promoted to Principal Medical Officer and is today remembered as the ‘father of the mini-pill’ for his role in reducing the oestrogenic content of oral contraception (Inman, 1999, Inman, 2006, Marks, 1999, Marks, 2002). Between 1967 and 1978, he was the government advisor chiefly responsible for deciding what action, if any, to take on HPTs.

Inman was also responsible for overseeing the ‘yellow card’ early-warning system of monitoring adverse reactions, so named for the distinctively coloured post-free business reply cards issued periodically to GPs and hospital doctors by the CSD. Doctors were encouraged to use the cards, which were arriving at a rate of around 1000 every month in 1964, to ‘report any suspected reaction to a new drug and any serious reaction to any drug, however old or new it was’ (Tansey and Reynolds, 1997: 118). The CSD did not have access to a computer, and Inman's ‘statistical calculations were worked with a slide-rule or log-tables and a hand- cranked “Facit” adding machine’ (Inman, 1999: 28). In the absence of robust baseline data, Inman developed a comparative method of assessing reactions caused by chemically similar drugs (Inman, 1999: 29).

The first drug that came to Inman's attention was a vasodilator that ‘quite obviously caused jaundice’. The company, Inman later recalled, was ‘persuaded to remove that voluntarily without any pressure. It was kept under wraps. There wasn't much publicity. We didn't seek any publicity’ (Tansey and Reynolds, 1997: 118). This was an ideal outcome for Inman, who preferred to resolve any potential safety issues quietly without involving doctors, the media or the general public.^6^ Owen Wade, Deputy Chairman of the Adverse Reactions Subcommittee in the mid 1960s, lamented this policy, which was intended to protect cooperative companies from bad press. Publicity, he later argued, would have ‘shown doctors the value of reporting adverse drug reactions and our reporting system would have got off the ground much quicker than it did’ (Tansey and Reynolds, 1997: 125). In reality, doctors voluntarily reported only a small fraction of the suspected adverse reactions they observed in clinical practice, perhaps only 10% for serious reactions (Rawlins, 1995). The fraction was even smaller for minor reactions and birth defects.

Inman later described the system's ‘inability to detect teratogenic drug effects’ as one of ‘several fundamental defects’ that had been ‘obvious’ from the start (Inman, 1999: 118). This is surprising for a system set up in response to thalidomide, a teratogenic drug, but several factors militated against detection. For one, gestation slowed down the already imperfect process of voluntary reporting. Noticing an adverse reaction 9 months after a drug had been prescribed – and in another patient, the child, who had not been given the drug directly – was a particular challenge as women went to gynaecologists, but took their children to paediatricians, and first-time mothers often moved house and changed doctors. Finally, the CSD insisted on the premarket testing of new drugs on pregnant animals, but this did not affect Primodos, an ‘old drug’ that was already on the market.

In 1970, the CSD was replaced by the Committee on the Safety of Medicines (CSM), a creation of the 1968 Medicines Act that set up a working party in 1976 to consider ways of ameliorating the early-warning system. However, at the same time, pressure was mounting to relax regulatory control. After the 1973 oil crisis, the pharmaceutical industry's export trade contributed even more significantly to the British Government's balance of payments and to maintaining the value of sterling abroad. When the Chancellor of the Exchequer was forced to obtain a conditional loan from the International Monetary Fund in 1976, the Government aligned its priorities even more with ‘those set by industry’ (Abraham and Davis, 2006: 141). Did the CSM eventually overcome the inability of its predecessor to detect another thalidomide? Inman did not think so. ‘Forty years after thalidomide’, he later wrote in his memoirs, there had been ‘no appreciable progress in the United Kingdom in the detection of drug-induced birth defects’ (Inman, 1999: 106).

## Pill scare and abortion panic

In 1965, when Isabel Gal began investigating spina bifida in Surrey, she struck up a correspondence with Inman that lasted many years. At the time, Inman privately agreed with Gal that, in view of the availability of non-invasive alternatives, HPTs were ‘not essential’ and it would ‘not be a disaster if [her] paper had the effect of reducing the frequency of their use’ (Inman, 1967a). However, he had doubts about her methodology and, as he later recalled, ‘there was another aspect that had to be absolutely taboo’ (Inman, 1999: 117). Namely, HPTs were compositionally similar to oral contraceptive pills, and Gal had implicated these in her letter to *Nature*: ‘A thalidomide-type scare in the media could easily cause panic among women using oral contraceptives. What would happen, for example, if a woman started taking the pill before she was aware that she was already pregnant?’ (Inman, 1999: 117).

What did Inman mean by this? Was he implying that such a woman would then want to have an abortion? Abortion became legal in Britain, but not Northern Ireland, in April 1968, when the Abortion Act 1967 came into effect (Sheldon, 1997). Inman continued, ‘On the one hand we had Dr. Gal's suspicion of a possible danger to the foetus and, on the other hand, a very real danger that publicity might cause a woman to stop using oral contraceptives or other preparations that were important in the treatment of a variety of gynaecological disorders. I was advised not to discuss these possibilities with anybody in case the idea that the HPT problem might have much wider repercussions inadvertently slipped out, though I can hardly believe that many people would not have thought of it themselves’ (Inman, 1999: 117).

Of Gal's report, a Schering executive claimed in an interview in 1980 that to ‘cast doubt on a major method of family planning, on this very slender evidence really, would not only have been a major commercial disaster but a real human disaster.. .we would have thrown panic into millions of women worldwide’ (Wintour, 1981). Inman later claimed that the ‘complexity of the situation made it very difficult for the Committee to undertake any action that would stop their use as a pregnancy test without compromising their other important uses’ (Inman, 1999: 118). However, was he more worried about preventing another thalidomide disaster or about preventing a thalidomide-type scare in the media?

Inman later claimed that, had Gal's study been more convincing, the CSD would have banned HPTs ‘immediately’ (Inman, 1999: 120). However, instead of taking immediate action, he launched a pilot study on drug-induced birth defects in collaboration with the medical division of the Office of Population Censuses and Surveys, a forerunner of the Office for National Statistics. The idea was to generate more robust epidemiological evidence for or against teratogenicity. As he later admitted, however, it took years to ‘assemble sufficiently large groups of children with each type of abnormality to allow valid conclusions to be drawn’, and progress was ‘lamentably slow, largely because higher priority had been given to concurrent problems with oral contraceptives, asthma deaths, the Eraldin disaster and the lack of equipment and staff’ (Inman, 1999: 119).^7^

Of the three concurrent drug problems, the one concerning oral contraceptives is the most proximate and instructive here; it had a direct bearing on Inman's decision-making process in the case of HPTs. Just before Christmas 1969, news broke that the pill was suspected of causing potentially fatal blood clots in otherwise healthy, young women. Such concerns are nearly as old as oral contraception itself, but came to a head in Britain only when the CSD held what was supposed to be a confidential briefing with pharmaceutical companies to privately disclose Inman's unconfirmed suspicions regarding the oestrogenic component of the pill. As with the vasodilator that caused jaundice, Inman would have preferred to work closely with industry and to exclude doctors, patients and the press. This time, however, the news leaked within hours.

First the *Daily Express*, a conservative mid-market broadsheet that had come out in favour of abortion law reform (Bingham, 2009: 88), then other newspapers and television programmes reported on the possible risk (Tansey and Reynolds, 1997: 121). Media exposure forced the CSD to hurriedly issue a yellow warning, its ninth, but the damage had already been done. Dozens of aggrieved GPs complained in the *BMJ* that the Committee's ‘maladroit’ and ‘unpardonable discourtesy’ had revealed the Government's ‘apparent contempt’ for doctors, some of whom had first learned of Inman's concerns from ‘agitated’ patients (Anon., 1969b). A lead article in the *BMJ* criticized the CSD for withholding the ‘statistical and clinical basis for its advice’ and went on to underscore the ‘lesson’ at hand, namely, ‘that official committees set up to inform the medical profession should communicate their information simply and solely to the profession. Where matters of life and death are concerned, as they are with the ‘pill’ and have been in several of the committee's previous reports, a press conference is an entirely inappropriate means of expression of the committee's views. The committee was not set up to educate – let alone alarm – the public’ (Anon., 1969a).

Inman's bruising experience with the pill, not to mention the then pervasive culture of medical paternalism that generally kept women in the dark about risky hormones and other gendered medical interventions (Tuana, 2006), helps to explain his reluctance to publicize concerns about HPTs. When the CSD finally issued a yellow warning in 1975, it was only after the *Sunday Times* – the same paper that campaigned on behalf of thalidomide victims – intervened (Gillie, 1975). Inman responded to the *Sunday Times* report by drawing a direct comparison in the *Guardian* between the ‘100,000 unwanted babies’ allegedly caused by the press leak in 1969 and the ‘very real danger’ that women would now be ‘pestering their doctors for an abortion’ (Pallister, 1975). The headline, ‘Doctor says drugs publicity could start abortion panic’, made explicit what had been taboo only a few years earlier.

HPTs, however, remained on the market for the treatment of menstrual disorders, and doctors continued to use them as pregnancy tests. From peak use by an estimated 100,000 women in 1971, prescriptions by one reckoning fell to around 40,000 in 1975, 25,000 in 1976 and 6000 in 1977 (Gillie, 1978).^8^ Schering added a red label to Primodos contraindicating pregnancy, but doctors did not see the packaging; they just wrote prescriptions (Anon., 1977b). Some pharmacists were uncomfortable dispensing Primodos but did so anyway, under the assumption that products presenting a similar risk to thalidomide had ‘automatically been withdrawn’ (Leddy, 2017). The British Pregnancy Advisory Service condemned the continued use of HPTs in February 1978 as an ‘area of persistent malpractice which represents an easily avoidable hazard’ (Brewer, 1978), and the *Sunday Times* was able to obtain the drug ‘on prescription’ in April, months after it was voluntarily taken off the British market by Schering for ‘commercial reasons’ (Gillie, 1978). By then, Jack Ashley, the Labour MP and deaf campaigner for disabled people who had cut his teeth on thalidomide, was calling for a public inquiry (Anon., 1977a).^9^

## The control mothers

By April 1975, when Inman reported some preliminary results to the *BMJ*, he and his colleagues had retrospectively examined only 149 abnormal pregnancies and the same number of normal controls: Primodos and related products ‘had been used by twenty-three mothers of abnormal babies compared with only eight of the controls’ (Greenberg et al., 1975). This finding, they contended, ‘tended to support’ Gal's conclusion, but Inman continued to suspect that the ‘reason for doing the test, such as the previous birth of an abnormal baby, would eventually prove to be the important factor and not the test itself. A woman who had already born an abnormal child was much more likely to bear another one’ (Inman, 1999: 119).

Inman had hoped to include at least 2000 cases and the same number of controls in his study every year, but underfunding and understaffing limited it to no more than 836 babies and the same number of controls, collected over several years. In the end, he concluded that underlying factors, not Primodos, were to blame: ‘As we had suspected, four times as many mothers of abnormal children than “control” mothers had a family history of previous abnormalities or had themselves borne abnormal children in the past. This was more than enough to account for the small excess of HPT use we had noted in our earlier report’ (Inman, 1999: 120).

But who did Inman imagine the control mothers to be? Were they women who had no pregnancy test whatsoever, or were they women whose urine had been tested? By the 1970s, pregnancy testing was increasingly the norm, and all kinds of women went to their GPs with a missed period (Olszynko-Gryn, 2017a). We know that some doctors preferred to order a urine test, while others sent their patient home with tablets. But what do we know about the women?

First-hand accounts suggest that patients given HPTs were similar to patients who had urine tests (several had both), and that many were first-time mothers with no prior history of pregnancy problems or malformed children. One such woman was 20 years old and living in the Midlands when she became pregnant in 1970. Her periods had ‘always been regular’ and she was ‘so excited’ at the prospect of pregnancy. She went ‘blindly’ to the GP who ‘prescribed two tablets, Primodos, to be taken over the two days and said it would cause a bleed if I wasn't pregnant’. She was, as she later recalled, ‘young, the first of all [her] friends to be pregnant and didn't even question it.. .’ (Collings, 2017).

A North Londoner, also in 1970, suspected pregnancy and ‘so went to [the] GP for confirmation’: ‘She gave me two pills to take, one to take straight away and the other 12 hours later. The pills were Primodos, and I distinctly remember asking the doctor that, if I was pregnant, would the pills hurt the baby at all as I said I would not take them if there was any chance that they could, but she reassured me that no harm would come to the baby. My period did not start so a week or so later she sent me to the hospital for the routine urine test which obviously confirmed that I was pregnant’ (Mills, 2017). The evidence is anecdotal, but it accords with what we know about the GPs who prescribed HPTs, namely, that they initially perceived the drugs chiefly as a convenient alternative to the laborious toad test, and later continued to use them out of habit.

Back in 1967, Gal's preliminary report had presented Inman with a ‘rather awkward problem’. On the one hand, he was not convinced of the validity of the data ‘on the grounds that the selection of cases was wrong’. On the other hand, he was not prepared to ‘rule out the possibility altogether’ (Inman, 1967b). He imagined getting to the bottom of things with a ‘prospective study of the outcome of pregnancy of matched pairs of women obtained from the same catchment area, one of each pair having had hormonal and the other biological pregnancy tests’. This would have involved a large number of doctors, and the decision on which test to apply would have been made randomly. However, the CSD lacked ‘facilities for further investigation’ and no such study was ever launched. Instead, Inman merely hoped that the manufacturers – ‘in view of the unreliability of hormonal pregnancy tests and of doubts about their safety, and of the dubious profitability of these products’ – would voluntarily ‘cease to promote them when and if the Gal paper is finally published’ (Inman, 1968).

In 1975, Inman conceded that the CSM was ‘defenceless in the matter of the eight-year delay’ between Gal's report and the first yellow warning that followed the publication of preliminary results in the *BMJ* and the *Sunday Times* exposé (Inman, 1975). Two years later, when the completed study was published, he and his co-authors presented the final results as ‘consistent with a general teratogenic effect of HPT’. The observed difference between case and control use of HPTs remained significant even when all ‘case mothers’ with a personal or family history of congenital malformation were removed from the analysis. The report, which was also published in the *BMJ*, agreed with the international consensus that had formed: ‘The excess use of HPT by case mothers found by us was not great and the association with malformations nonspecific; alternative risk-free methods of pregnancy diagnosis are, however, available and the use of HPTs is unnecessary’ (Greenberg et al., 1977).

Some 20 years later, however, Inman felt vindicated that the statistical correlation between the use of HPTs and malformations could be explained in terms of underlying factors. Standing by the policy of inaction that had prevailed in the 1970s, he wrote in his memoirs: ‘It is the coward's way out to take action and perhaps bask in the reflected glory of the newspapers for doing something positive against a drug. It takes more courage to exercise restraint, and I vigorously defend my colleagues and former colleagues on the Committee and in the Department of Health in taking no action at that stage’ (Inman, 1999: 124). What had changed? The answer, in part, lies in a German study that played a crucial role in blocking Jack Ashley's call for a public inquiry.

## The German study

On 26 May 1978, Jack Ashley and Labour Health Minister Roland Moyle debated the need for an independent public inquiry into Primodos in the House of Commons. Ashley contended that in view of Gal's original warning in 1967 and of the ‘gravity of severe congenital abnormality’, HPTs should have been ‘immediately suspended pending full clearance by the committee’. This should have been ‘axiomatic and automatic, especially after our experience of the thalidomide tragedy. Instead, hesitancy was compounded by incompetence and, as a result, more than 1,500,000 pregnant women were placed at unnecessary risk by being given these drugs and thousands of children may have been gravely damaged’. Ashley sought an inquiry to ‘establish why the Government failed’ and pressed Moyle on whether he accepted that studies ‘conclusively prove that HPT drugs sometimes – not necessarily always – cause abnormalities. Does he confirm or deny that?’ (Ashley, 1978).

‘Until today’, Moyle claimed, his answer ‘might have been “Yes”’. However, on the morning of the debate, he had acquired ‘some evidence of testing in this field by the German Research [Foundation]’. Planned in response to thalidomide and carried out in 21 hospitals in West Germany from 1964 to 1974 (Michaelis et al., 1983), this prospective study was, according to Moyle, ‘the most comprehensive investigation ever conducted’ on the suspected teratogenic effects of drugs administered in early pregnancy; it ‘covered nearly 15,000 women, and nearly 8,000 of the tests on those women have been evaluated in the preliminary report’. The results, Moyle continued, did ‘not provide evidence that hormonal pregnancy tests were harmful. The study shows that many other factors can influence the outcome of pregnancy. For example, women with abnormal babies had had, according to the study, more previous miscarriages, had had more abnormal children and had suffered more frequently from chronic diseases of various kinds. Cigarette smoking was shown to have an unfavourable effect’ (Moyle, 1978b).^10^

The German study, Moyle argued, revealed that ‘anxious’ women with a history of miscarriage ‘tended to make greater use’ of HPTs and that this supported the ‘view that the results of the committee's studies on hormonal pregnancy tests may have been due to some other unidentified confusing factor, most likely relating to the reason that the pregnancy test was used’. As for the parents seeking compensation, they would have to ‘get medical research done’ to provide a ‘causal connection between the application of these tests and the damage that was caused’, and that connection did ‘not exist at present’ (Moyle, 1978b).

Causality is notoriously difficult to establish (John, 2010), and Moyle did not elaborate on how the parents were supposed to finance or otherwise support the medical research. When asked on ITV's ‘The London Programme’ whether, given that harmless alternatives existed, it would ‘not have been perhaps wiser for the [CSM] back in the late 1960s to have taken some action’, he impatiently responded, ‘Well that's all very much water under the bridge. Within 1971 they were still on the market when the Medicines Act became law and of course if [.. .] the Department were to withdraw something from the market at that stage they had to show that it was positively dangerous or unhelpful or something of that sort and of course they didn't have the evidence to do that, so they couldn't’ (Moyle, 1978a).

For journalist Greg Dyke, who produced the programme, it was ‘clear’ that Moyle's ‘advisers in the Department of Health’, particularly those involved with the CSM, were ‘strongly opposed to any form of independent public inquiry’ (Dyke, 1978). Inman later claimed that he ‘could not understand the arguments against such an enquiry other than the cost to the taxpayer’ (Inman, 1999: 121). However, at the time, it was he who furnished Moyle with the German study.

The German study was Moyle's trump card. However, the findings that Inman had fed Moyle in advance of the debate were only preliminary. When the final report was published in 1983, the results were less conclusive than Moyle had made them out to be in 1978. Singling out Duogynon (Primodos) as particularly troublesome, the German team concluded: ‘The interpretation of the Duogynon analysis seems to us less evident than for the antiemetic drugs [to treat morning sickness] and Proluton [mainly to prevent miscarriage]. Although we did not find a significant association, the observed odds ratios were greater than 1, and their upper confidence limits were rather high, which could be regarded as being in accordance with the positive findings of other studies. The lack of significance could then be interpreted as due to the small number of observations. We therefore consider it as adequate that general consensus was obtained not to use Duogynon during pregnancies’ (Michaelis et al., 1983: 64).^11^

This is not a smoking gun that Duogynon/Primodos definitely caused birth defects. However, it falls well short of establishing harmlessness. If anything, it tended to support, not undermine, the ‘positive findings’ that came before.

## Norway and other countries

Duogynon was finally taken off the German market in 1981. However, renamed ‘Cumorit’, it continued to be used informally as an abortifacient in developing countries (Bonnema and Dalebout, 1992, Ujah, 1991). The final report of the CHM's EWG notes that in ‘different European countries and globally, decisions to withdraw HPT products were taken in a staggered and uncoordinated way’. It goes on to explain this in terms of the complexity of the market, ‘differences of opinion on the strength of the evidence for an association between HPTs and congenital anomalies’, and the lack of well-developed ‘communication channels between regulators in different countries’ (Commission on Human Medicines, 2017: 92). In this section, we briefly survey the warnings that led to a ban in some countries earlier than in others. In addition to the factors noted by the EWG, we explain the decision to take action in Norway and other countries in terms of the international consensus that, in light of a non-invasive alternative, HPTs were unnecessary and so could be taken off the market without harming patients. We also find that Inman's preoccupation with protecting the market for oral contraception and preventing an abortion panic was not widely shared outside Britain.

Norway, which established one of the first drug regulatory systems in 1928, provides the most striking and instructive contrast. In line with the sociodemocratic principles of the Norwegian welfare state, pharmaceutical products were judged more on the basis of expert evaluation than on commercial potential (Pedersen and Lie, 2013). Between 1938 and 1994, when Norway joined the European Economic Area, its drug policy was based not only on safety, efficacy and cost, but also on medical need. The country's Medical Need Clause (MNC) required any new drug to meet a clear-cut therapeutic need and to represent an improvement over alternatives already on the market. Put into action, the MNC effectively restricted the number of drugs on the Norwegian market in the 1970s to around 2000, which was far fewer than the number in most other European countries (7000–25,000). Finally, the Norwegian Medicines Agency (NoMA) subjected all new drugs to a probationary approval period of 5 years followed by a re-assessment process that could result in de-authorization (Brooks and Geyer, 2016).

As apparently non-essential products for which harmless alternatives existed, HPTs were in a comparatively vulnerable position when Bergen gynaecologist Per Bergsjø wrote against them in the *Journal of the Norwegian Medical Association* in July 1968: ‘The consequence of [Gal's letter in *Nature*] must be that we abandon the so-called HPTs. Even though there are some doubts around the validity of the findings, the fact that this diagnostic method is so uncertain is in itself a reason for not using it. Today there are more direct and completely harmless methods for diagnosing pregnancy’ (Bergsjø, 1968; translation by E. Bjørvik).

NoMA's Specialist Control Board supported Bergsjø's recommendation. Echoing the wording of Bergsjø's letter, the Board notified Schering's Norwegian distributor of their decision in February 1970: ‘Today there are more direct and completely harmless diagnostic methods to diagnose pregnancy. The subject has been discussed in a meeting in the Specialist Control Board, which decided that the indication ‘pregnancy test’ hereafter shall not be approved for hormone-based drugs’ (Wold, 1970; translation by E. Bjørvik).

A Norwegian study linking HPTs to hypospadias, a congenital malformation of the penis, added urgency to the decision. Bergen paediatrician Dagfinn Aarskog wrote to NoMA and argued in *Acta Paediatrica Scandinavica* in June 1970 that even ‘circumstantial evidence [.. .] should exhort to caution in giving such drugs to pregnant women. [.. .] With the simple laboratory methods now at hand to test for pregnancy, there is no need for these potent steroids to be used for this purpose’ (Aarskog, 1970: 35). NoMA cancelled the indication of ‘pregnancy testing’ in September 1970 and, by August 1972, the entry in the Norwegian pharmaceutical industry's drug catalogue explicitly warned against the use of Primodos in pregnancy on the grounds that it could cause fetal virilization (Koller, 1972). Primodos remained available for the treatment of amenorrhoea until 1973, when NoMA reclassified the drug as ‘not medically justified’ and it was taken off the market with compliance from Schering (Barfods Farmaceutiske, 1973).

In Finland, Primodos tablets were discontinued when the initial 5-year licence came up for renewal in 1971 and was rejected. Injections remained available for use in the treatment of amenorrhoea until 1978, when they were taken off the market by the distributor (Weßel, 2018). Following the independent corroboration of Aarskog's findings by research at the Karolinska Institute in Stockholm, the Swedish Medical Board removed the indication of ‘pregnancy testing’ from the entry for Primodos in Sweden's national drug registry in 1972, although Primodos and Duogynon continued to be used in Sweden for the treatment of amenorrhoea until 1975 and 1978, respectively. In France, where pregnancy testing was linked to illegal abortion until the law changed in 1975 (Cahen, forthcoming), products such as Primodos were viewed with suspicion and were not authorized for use as HPTs. Lack of authorization, however, did not prevent their off-label use in pregnancy testing until the mid 1970s, and some brands persisted on the market as treatments for amenorrhoea until more recently.

Australia, New Zealand and the USA all took action in 1975. The Australian Government took action immediately after William Brogan, a paediatrician investigating cleft palate in Western Australia, warned against HPTs in the *Medical Journal of Australia* in January 1975 (Brogan, 1975, van den Heuvel, 1975). New Zealand took the additional step of recalling stock from pharmacy shelves (Medsafe, 2017). In the USA, where HPTs were used informally as ‘morning-after’ contraceptives (Anon., 1967), the Food and Drug Administration (FDA) banned them at around the same time (Kazmierski, 1976: 4–5). In contrast to Britain, the regulators in these countries do not appear to have been concerned about a pill scare or abortion panic. On the contrary, the FDA's strongly worded ‘warning on use of sex hormones in pregnancy’, which also covered diethylstilboestrol (DES),^12^ explicitly stated: ‘if pregnancy is suspected in a patient receiving oral contraceptives, these should be discontinued immediately. Obviously, every effort should be made to assure that a woman is not pregnant before prescribing sex hormones for any purpose.’ As for HPTs, the FDA's Ob-Gyn Advisory Committee ‘concluded that the risk of teratogenicity also precludes use of those hormones as a diagnostic test for pregnancy’ (Anon., 1975: 4).

## Towards closure?

On 21 February 2018, the British Prime Minister Theresa May ordered another review of HPTs, this time alongside vaginal mesh implants, a treatment for incontinence linked to chronic pain, and sodium valproate, an epilepsy drug known to cause birth defects (a risk about which pregnant women were inadequately informed). Led by Baroness Julia Cumberlege, it will ask whether there needs to be a ‘public inquiry [.. .] into any of the cases’ (Triggle, 2018), all three of which involve gendered medical interventions specifically affecting women and government responses that have left campaigners aggrieved. ACDHPT, meanwhile, is calling for a full judicial or independent review of all the evidence, including archival records that they (and Sky News) believe point to a cover up.

Such a review is warranted, we believe, not least because the regulatory process in Britain was clearly influenced by nationally specific and even idiosyncratic factors that were quite independent of the old scientific data examined by the EWG. Inman, for example, prioritized averting a thalidomide-type pill scare at a time when oral contraception was an economically important drug, the highly publicized risk of thrombosis was threatening an abortion panic, and medical paternalism generally militated against informed consent. In contrast to Britain, regulators in Norway and other countries took a more precautionary line starting in 1971, even though they were working with a similarly anecdotal or inconclusive evidence base. By 1975, an international consensus had formed that HPTs were redundant because of the widespread availability of non-invasive alternatives (urine tests). Medical advisors in other countries were seemingly unconcerned that taking HPTs off the market would cause a pill scare, abortion panic or ‘human disaster’. The alarms sounded by Isabel Gal and by her Norwegian, Swedish and Australian counterparts were heeded by governments around the world.

This article has just scratched the surface of a much bigger and even more international story about HPTs, sex hormones, and the contested use and regulation of drugs in pregnancy. In the absence of fine-grained data, there is much that will necessarily remain obscure, but an independent review of all the available evidence could significantly extend the analysis we have presented here. A more comparative perspective than was achieved by the CHM's EWG could further contextualize the British regulatory process in relation to parallel developments in other countries where medical advisors and governments took divergent lines based on similar knowledge. A fuller account, not limited to the scientific case for or against teratogenicity, would have the potential not only to bring a form of closure to the families who believe that they were affected by HPTs, but also to shed light on pressing issues of more general significance regarding risk, regulation and communication between policy makers, medical experts and patients. MHRA would have much to learn from such an account about how the regulatory process worked – or failed to work – in the past, and about how it can be improved in the future.

## Footnotes

1 Anomalies investigated in connection with HPTs include neural tube defects, heart defects, limb defects, cleft palate and genital abnormalities.

2 On the more general point that ‘weak evidence should be taken into account when the costs of error [. . .] are potentially very high’ (Lewens, 2015: 160).

3 Recent experimental results implicate the components of Primodos as ‘potentially teratogenic, depending on dose and embryonic stage of development in the zebra fish’ (Brown et al., 2018: 1).

4 One tablet of Anovlar contained 4 mg norethisterone acetate, a progestin, and 0.05 mg of ethinyloestradiol, an oestrogen (Marks, 2001: 77); one tablet of Primodos contained 5 mg norethisterone acetate and 0.01 mg ethinyloestradiol (Brown et al., 2018).

5 Thanks to Isabel Davis (Birkbeck, University of London) for this point.

6 On the active production and maintenance of ignorance, including by medical and scientific experts (Proctor and Schiebinger, 2008).

7 On Eraldin, also known as practolol (Abraham and Davis, 2006). On asthma deaths (Pearce, 1996, Pearce, 2007). Practical problems (funding constraints, coordination, sufficient population samples) have been regarded as difficult to overcome, even decades after the establishment of birth defect monitoring systems such as EUROCAT (Dolk, 2005).

8 For a discussion of the available quantitative data, see Commission on Human Medicines (2017: 17–19).

9 On Ashley's life and career (Ashley, 1973, Ashley, 1992). Ashley was also a key player in concurrent campaigns for vaccine-damaged children (Millward, 2017).

10 On the construction of smoking during pregnancy as a public health concern and social problem in the 1970s (Oaks, 2001).

11 Commonly used in case–control studies, odds ratios measure associations between exposures and outcomes (Szumila, 2010).

12 The oestrogen analogue DES was used for decades to prevent miscarriage, and was used in the 1960s as a ‘morning-after’ pill until research linked it to a rare form of vaginal cancer in girls who had been exposed in utero. For a recent historical analysis, see Gaudillière (2014).
